# Treatment of mucosa associated lymphoid tissue lymphoma with a long‐term once‐weekly regimen of oral azithromycin: Results from the phase II MALT—A trial

**DOI:** 10.1002/hon.2555

**Published:** 2018-09-19

**Authors:** Heimo Lagler, Barbara Kiesewetter, Werner Dolak, Markus Obermueller, Ingrid Simonitsch‐Klupp, Julius Lukas, Ortrun Neuper, Wolfgang W. Lamm, Marius E. Mayerhoefer, Markus Raderer

**Affiliations:** ^1^ Department of Medicine 1, Division of Infectious Diseases and Tropical Medicine Medical University of Vienna Vienna Austria; ^2^ Department of Medicine 1, Division of Oncology Medical University of Vienna Vienna Austria; ^3^ Department of Medicine III, Clinical Division of Gastroenterology and Hepatology1 Medical University of Vienna Vienna Austria; ^4^ Department of Pathology Medical University of Vienna Vienna Austria; ^5^ Department of Ophthalmology Medical University of Vienna Vienna Austria; ^6^ Department of Biomedical Imaging and Image‐guided Therapy Medical University of Vienna Vienna Austria

**Keywords:** azithromycin, clinical trial, MALT lymphoma

## Abstract

The macrolide clarithromycin has been reported as active for therapy of mucosa associated lymphoid tissue (MALT) lymphoma. Pharmacokinetic properties, however, require continuous daily intake over a prolonged period of time. As the macrolide azithromycin is characterized by a long half‐life as well as potential antineoplastic activity in vitro, we have performed a phase II trial of long‐term once‐weekly oral azithromycin for treatment of MALT lymphoma. In a 2‐stage‐design, 16 patients (10 f/6 m) with histologically verified and measurable MALT lymphoma were included in the first phase of the trial, which could be expanded to a maximum of 46 patients depending on remissions in the first phase. Patients were given oral azithromycin 1500 mg once‐weekly 4 times a month, and restaging was performed after 3 and 6 months. Two patients had gastric and 14 extragastric MALT lymphoma; 12/16 patients were treatment‐naive and received azithromycin as first line treatment. Tolerance of this regimen was excellent, and 14/16 patients received 6 months of treatment as scheduled, while 1 patient each discontinued after 4 (progressive disease) and 1 cycle (personal reasons), respectively. The most commonly observed side effects were mild nausea (n = 8) and diarrhea (n = 4). Efficacy, however, was low as only 4/16 patients (25%) responded, with 2 complete and 2 partial remissions, 9 patients (56%) had stable disease, and 3 patients 19%) were rated as progressive disease. As the predefined activity of more than 7/16 patients responding was not reached, the study was stopped after 16 patients. Although long‐term once‐weekly oral azithromycin showed some antilymphoma activity, the response rate was below the predefined threshold of interest. However, based on our data, one cannot rule out suboptimal dosing in our study; attempts to study azithromycin at a different mode of application might be warranted in the future.

## 1 INTRODUCTION

Extranodal marginal zone B‐cell lymphoma of the mucosa associated lymphoid tissue (MALT) is an indolent disease that accounts for roughly 8% of all newly diagnosed adult lymphoma cases.^1^ Owing to the role of chronic infections^2^, ^3^, ^4^ and autoimmunity^5^, ^6^ in the pathogenesis of the disease, antibiotic therapy as well as immunomodulatory approaches have repeatedly been tested. Especially in patients with documented infection with *Helicobacter pylori* (HP) and—to a lesser extent due to the wide geographic variation of infection rates—*Chlamydophila psittaci*, antibiotic therapy is the treatment of choice for gastric and also early stage ocular adnexal MALT lymphoma resulting in response rates ranging between 45 and 75%.^4^

The macrolide antibiotic clarithromycin has recently been reported as an active agent for therapy of patients with MALT lymphoma. While initially having been included in most regimens applied for eradication of HP, the increasing rate of resistance to clarithromycin of various HP strains is becoming a problem. While a small study in Austria has shown the rate of clarithromycin‐resistant HP strains to be roughly 13% in MALT lymphoma patients,^7^ the small number of patients studied along with the fact that the overall resistance rate is among the highest in Europe has led to the recommendation that clarithromycin should no longer be used in a triplet HP‐eradication therapy, while it is still recommended as part of quadruple therapies according to the Maastricht V consensus.^8^, ^9^

Emerging results have shown a surprisingly high direct antineoplastic activity of the macrolide in patients with MALT lymphoma, including heavily pretreated patients. In a recent paper including 55 patients, a response rate of 47% with a 3‐year progression free survival of 55% was reported The median number of prior therapies was 2 (range; 1‐5), and there was no difference between 1 g/d versus 2 g/d in terms of efficacy.^10^ The mode of application, however, requires good patient compliance, as clarithromycin is most commonly given orally over 6 consecutive months at a daily dose of 2 × 500 mg.

As opposed to this, the macrolide azithromycin appears to be characterized by a prolonged terminal systemic half‐life of 68 hours as compared with 5 hours for clarithromycin.^11^ It has further been shown that azithromycin reaches high tissue concentrations especially at inflammatory sites and even higher in macrophages and leukocytes (more than 100‐fold serum concentration)^12^, ^13^, ^14^ and therefore has the potential advantage of intermittent dosing. In addition to in vitro data suggesting azithromycin to be a potent mTOR inhibitor in CD4+ T‐cells and thus also displaying immunomodulatory properties,^15^ different high‐dose long term applications have been shown to be safe in adults and children with cystic fibrosis (10 controlled studies including 632 patients receiving azithromycin over 2‐12 months).^16^ In view of this, we have hypothesized that azithromycin might have efficacy in MALT lymphoma and have performed a phase II trial to assess the activity of the drug.

## 2 PATIENTS AND METHODS

The MALT‐A protocol had been approved by the Ethical Board of the Medical University of Vienna and had been registered at http://www.clinicaltrials.gov before initiation. Patients older than 18 years with a histologically verified MALT‐lymphoma according to the recent WHO Classification^1^ were eligible. In case of gastric origin, the disease had to be at least refractory to HP eradication as judged by a minimum follow‐up time of 12 months after initial antibiotic therapy, while patients with nongastric MALT‐lymphoma were directly eligible. In addition, patients with relapses from prior chemoimmunotherapies or radiation were also eligible after a minimum of 4 weeks after the last dose.

Patients had to have radiologically measurable disease or in gastric MALT lymphoma disease amenable to histological response assessment using the Groupe d' Etude des Lymphomes de l' Adulte (GELA) criteria.^17^ In case of radiologically measurable disease, the standard Response Evaluation Criteria In Solid Tumors (RECIST) criteria for complete remission (CR), partial remission (PR), stable disease (SD), and progressive disease (PD) were applied, while response assessment of MALT lymphoma restricted to the stomach was based on the histological assessment of rebiopsies according to the GELA criteria as outlined by Copie‐Bergman et al.^17^ Additional inclusion criteria were adequate hematological, renal, and hepatic function.

Known allergy/hypersensitivity to macrolides, cardiac conditions including unstable angina, acute myocardial infarction within 6 months prior to randomization, congestive heart failure (NYHA III‐IV), arrhythmia unless controlled by therapy, with the exception of extra systoles or minor conduction abnormalities, and long QT syndrome (QTc interval > 450 ms) served as exclusion criteria. Concomitant medication with theophylline, ergotamine, and digitalis was not allowed, and any investigational drug employed before inclusion had to have been discontinued for more than 28 days before the first dose of azithromycin. Patients with active opportunistic infections, HIV positivity, active psychiatric disorders, and histology results other than MALT‐lymphoma and another malignancy other than squamous cell carcinoma, basal cell carcinoma of the skin, or carcinoma in situ of the cervix within the last 3 years were excluded, as were individuals with major surgery, other than diagnostic surgery, within the last 4 weeks.

Azithromycin was given orally at a dose of 1500 mg once weekly every 7 days during 4 weeks (=1 cycle). This dose was based on the only long‐term experiences published in patients with cystic fibrosis, as this dose had been shown as safe for long term use.^16^ Before therapy, all patients underwent imaging of the respective target organ using sonography, computed tomography, or magnetic resonance imaging as indicated plus imaging of thorax/abdomen, in case of gastric lymphoma, gastroscopy with multiple biopsies was performed. Positron emission tomography‐computed tomography or positron emission tomography‐magnetic resonance imaging could be performed at the discretion of the investigator. Restaging was performed after 3 cycles, and in case of SD or better response according to RECIST‐guidelines 1.0 for nongastric and GELA criteria for gastric MALT lymphoma, therapy was continued for another 3 courses up to a maximum of 6 cycles. Restaging was again performed after 6 courses, and the best response to treatment in each patient was recorded.

Side effects were recorded every 4 weeks, and patients were provided with study drug for the following cycle during these control visits. The study drug azithromycin (Zithromax® 500‐mg tablets, Pfizer) was bought for application in this trial.

The study was planned according to a 2‐stage design, with an interim analysis after the first 16 patients (phase I) for efficacy and safety. In case of an objective response in a minimum of 8 patients (ie, 50%), the drug would be considered active (alpha 0.050, beta 0.02) and another 30 patients for a total of 46 patients would be enrolled for more exact definition of efficacy. In case of 7 or less patients responding, the study would be discontinued. This assumption of interest was based on response rates reported for clarithromycin in the current literature.^10^, ^18^

Primary endpoint was the rate of objective responses as judged by best response, ie, CR, PR, SD/no change, and PD as defined by RECIST criteria version 1.0 in terms of extragastric MALT lymphoma or GELA criteria for histologic response in gastric MALT lymphomas.

Additional endpoints were safety and tolerance of treatment in terms of hematologic and nonhematologic side effects as assessed by the investigators. All patients eligible for the study and receiving at least 1 dose of study drug were included in the analysis. Patients who dropped out or died prior to the first response assessment were included in the denominator when calculating the response rate. Patients enrolled into the study but who received no study medication were excluded according to the protocol.

## 3 RESULTS

A total of 16 patients were enrolled into the first phase of the study according to protocol. The majority of patients (n = 10) were female, while 6 were male, with the median age being 68.3 years (interquartile range 17.2). For patient characteristics, see Table 1.

**Table 1:** MALT lymphoma patient characteristics (n = 16) and study results

No	Sex (m/w)	Age (Years)	MALT Location	LN Involvement	Previous Therapy	W12	W24	W36	EOS
1	m	74	Stomach	No	‐	SD	SD	SD	SD
2	w	57	Orbit, subcutaneous	No	‐	SD	SD	SD	SD
3	w	78	Orbit	No	‐	SD	SD	SD	SD
4	m	76	Orbit	No	‐	SD	SD	SD	SD
5	w	88	Adrenal gland, breast	Yes	Chlorambucil	SD	PD	—	PD
6	m	59	Lung	Yes	‐	PR	PR	PR	PR
7	m	47	Orbit	No	‐	CR	CR	CR	CR
8	w	71	Lung	Yes	‐	SD	SD	SD	SD
9	m	57	Stomach	Yes	Rituximab Bendamustine	PD	‐	‐	PD
10	w	67	Orbit	No	‐	SD	SD	SD	SD
11	m	76	Parotid gland	Yes	‐	SD	CR	CR	CR
12	w	64	Orbit	No	R‐CHOP clarithromycin	SD	SD	SD	SD
13	w	65	Breast, subcutaneous	No	Lenalidomide Rituximab	‐	‐	‐	‐
14	w	79	Orbit	No	‐	SD	SD	SD	SD
15	w	49	Parotid gland, orbit	No	‐	SD	PR	PR	PR
16	w	68	Orbit	No	‐	SD	SD	SD	SD

Abbreviations: MALT, mucosa associated lymphoid tissue; LN, lymph node; W, week; EOS, end of study; SD, stable disease; CR, complete remission; PR, partial remission; PD, progressive disease; R‐CHOP, rituximab, cyclophosphamid, hydroxydounorubicin, oncovin, prednisolone.

In total, 9 patients (56%) had MALT lymphoma originating in the ocular adnexa; 2 patients each had lymphoma of the lung, the stomach, and the breast, respectively; while 1 had parotid and subcutaneous MALT lymphoma each. The 2 patients with gastric MALT lymphoma had undergone unsuccessful antibiotic therapy for HP initiated immediately upon diagnosis, although they were then found to be negative using histology and serology. Four patients had relapsed following prior systemic therapy, while 12/16 patients (75%) were treatment naive.

Tolerance of treatment was good, with 14/16 patients undergoing a full 6 months of treatment as scheduled. One patient was taken off study after 4 courses due to PD, while 1 patient discontinued therapy in the absence of relevant side effects for personal reasons after 1 cycle and was consequently rated as PD in the final analysis.

Toxicities were mainly mild and mostly unspecific, with nausea grade I in 8, grade II in 3 patients, and grade III in 1 patient; emesis grade II in 1 case, and diarrhea grade I in 4 and grade II in 2 patients and gastrointestinal complaints (including flatulence, bloating, and cramps grade I/II) in a total of 6 patients (Table 2). No cardiac complaints of any grade were reported.

**Table 2:** Tolerability of long‐term once‐weekly oral study medication azithromycin at 16 MALT lymphoma patients

Grade	Adverse Event	n	%
3	Nausea	1	6
2	Nausea	3	19
	Diarrhea	2	13
	Chills	1	6
	Dyspepsia	1	6
	Dyspnea	1	6
	Emesis	1	6
	Fatigue	1	6
	Gastrointestinal/digestive problems	1	6
	Headache	1	6
1	Nausea	8	50
	Diarrhea	4	25
	Emesis	2	13
	Fatigue	2	13
	Flatulence	2	13
	Gastric pain	2	13
	Headache	2	13
	Bloating	1	6
	Dry mouth	1	6
	Dyspepsia	1	6
	Gastric discomfort	1	6
	Gastrointestinal/digestive problems	1	6
	Joint pain	1	6
	Loss of appetite	1	6
	Vertigo	1	6
	Vomiting	1	6
	n = number of reported adverse events		

In spite of the excellent tolerance, only 4/16 patients (25%) responded (2 CR and 2 PR). An additional 8 patients (50%) had SD, 3 patients (19%) were rated as PD, and 1 patient transformed to gastric diffuse large B‐cell lymphoma and was consequently treated with R‐CHOP, resulting in CR. The rate of tumor control (CR, PR plus SD) was 12/16 (75%) at 6 months.

## 4 DISCUSSION

To the best of our knowledge, this is the first study to assess the clinical activity of the macrolide antibiotic azithromycin as an antilymphoma agent in MALT lymphoma. While clarithromycin has been reported as an active antineoplastic agent in multiple myeloma and MALT lymphoma, as well as having been shown to reverse resistance to lenalidomide‐containing therapy in multiple myeloma, only in vitro data have been generated so far for azithromycin. In addition to the antimicrobial activity, both agents have been shown to induce apoptosis of activated lymphoid cells by downregulation of BCL‐xL,^19^ and azithromycin has also been reported to be a potent inhibitor of T‐cell function via inhibiting m‐TOR.^15^ As T‐cells play an important role in the development and maintenance of MALT lymphoma in patients with infection‐ or autoimmune‐triggered disease, these in vitro data along with the prolonged half‐life allowing for convenient dosing have prompted us to initiate the phase II MALT‐A study.

Tolerance of treatment was excellent, with the most common side effects being mild nausea in 8 and diarrhea in 4 patients each (see Table 2) comparable with similar long‐term high‐dose regimes used in cystic fibrosis patients.^16^

Apart from a low rate of toxicity and the convenient mode of intake, however, the trial was stopped after 16 patients because the predefined thresholds of activity were not met. Based on data generated with clarithromycin, the assumption of comparable efficacy had led us to define 8 responses in the initial 16 patients as minimum for continuing into phase 2 of the trial. While azithromycin did display some antineoplastic activity, only 4/16 patients showed an objective response (2 CR and 2 PR), resulting in a response rate of 25%. In addition, 8 patients (50%) had SD and 2 PD, 1 patient transformed to diffuse‐large B‐cell lymphoma, and 1 patient discontinued therapy for personal reasons after 1 cycle (and was thus also rated as PD). Taken together, the rate of disease control (CR, PR, and SD) was 75% at 6 months of therapy.

While this was not a head to head comparison but rather a proof‐of‐principle phase II trial, the results reported with clarithromycin appear to be favorable compared with our data. Overall, a response rate of 47% (24% CR and 22% PR) was reported for clarithromycin, with a higher overall response rate in the subgroup with gastric lymphoma (78% vs 41%). The overall disease control rate was 76%, and the 3‐year progression free survival 55%. The most common side effect was nausea, which appeared to be more common with higher doses, and was encountered in 26% of patients undergoing therapy with 2 g/d but necessitated interruption of treatment in only 2/23 patients.^18^

There are different potential explanations for the apparently different antineoplastic efficacy of the 2 macrolides, suggesting that the antilymphoma activity might not be a class effect. First of all, one cannot rule out suboptimal dosing of azithromycin as used in our study based on data obtained for antibiotic activity. As azithromycin has the lowest bioavailability of the macrolide antibiotics at 37%^20^ compared with clarithromycin with 50%,^11^ the reason for the low response rate could be that drug levels were simply not adequate for a sufficient antilymphoma activity. Further attempts to study azithromycin at a different mode of application such as higher doses of extended release formulations^21^ might be tested. In addition, an intravenous application of azithromycin could also potentially circumvent the poor bioavailability.

However, based on in vivo data, at least the immunomodulatory effect of CD4+ cells was more pronounced even at low concentrations of azithromycin, while clarithromycin suppressed T‐cell function only at the highest dose investigated.^15^ As this effect has been reported to occur via inhibition of mTOR, which has also been applied as antineoplastic therapy in MALT‐lymphoma by using everolimus,^22^ a potentially higher activity of azithromycin might be expected even at lower concentrations.

In addition, owing to the biology of MALT lymphoma and in view of the often delayed onset of treatment effects with both HP‐eradication as well as other immunomodulatory therapies including ImiDs,^23^ the follow‐up period might have been too short to discover the optimal response. However, at least with clarithromycin, a delayed response was not reported, as all patients had already responded at the end of therapy.^18^

The collective of patients treated within our study and the patients reported with clarithromycin might also be different. In fact, Ferreri and coworkers have reported a better response rate in patients with gastric versus nongastric MALT‐lymphomas (78% vs 42%). As only 2 out of our 16 patients had gastric lymphoma, this might have biased findings to some extent, as could have the fact that the large majority of patients in our series were untreated as opposed to the salvage‐setting reported in the clarithromycin studies.^10^, ^18^, ^24^

In addition, the fact that macrolides exert their effects mostly via immunomodulation suggests that they might work best in MALT lymphoma developing in the background of chronic antigenic stimulation. While the rate of autoimmune‐diseases was not reported in the studies on clarithromycin, 36% of patients in the recent analysis by Ferreri had lymphoma related to chronic infection, while none of our patients was found positive for Hepatitis B or C, HP (both patients with gastric MALT lymphoma had been tested negative) or *Chlamydophila psittaci*, and only 1 had an underlying autoimmune condition (ie, SLE).

Taken together, azithromycin displayed some activity against MALT lymphoma in our series including 2 CR and 2 PR but showed a disappointing response rate overall and thus should not be used in clinical routine for treatment of such patients.
